# Cochlear Meniere's: A Distinct Clinical Entity With Isolated Cochlear Hydrops on High-Resolution MRI?

**DOI:** 10.3389/fsurg.2021.680260

**Published:** 2021-06-16

**Authors:** Jose E. Alonso, Gail P. Ishiyama, Rance J. T. Fujiwara, Nancy Pham, Luke Ledbetter, Akira Ishiyama

**Affiliations:** ^1^Department of Head and Neck Surgery, University of California, Los Angeles, Los Angeles, CA, United States; ^2^Department of Neurology, University of California, Los Angeles, Los Angeles, CA, United States; ^3^Department of Neuroradiology, University of California, Los Angeles, Los Angeles, CA, United States

**Keywords:** endolymphatic hydrops, cochlear Meniere's disease without vertigo, high-resolution MRI, cochlear hydrops, low-frequency hearing loss

## Abstract

**Objective:** Describe the clinical characteristics of patients with isolated cochlear endolymphatic hydrops (EH).

**Study design:** Clinical case series.

**Setting:** Tertiary Neurotology referral clinic.

**Patients:** All subjects presenting to a University Neurotology clinic during a 1-year period from July 2015 until August 2016 who had isolated cochlear EH on MRI. Patients with a history of temporal bone surgery prior to the MRI were excluded.

**Intervention:** High-resolution delayed-intravenous contrast MRI.

**Main outcome measures:** Audiometric and vestibular testing, clinical history analysis.

**Results:** 10 subjects demonstrated *isolated*, unilateral cochlear hydrops on MRI. None of these patients met the criteria for Meniere's disease. Mean age of the group was 66.4 years and most were males (70%). Unilateral aural fullness (70%), tinnitus (80%), and hearing loss (90%) were frequently observed. Only one patient presented with unsteadiness (10%) and one patient had a single isolated spell of positional vertigo 1 month prior to the MRI (10%) but no further vertigo spells in the 4 years following the MRI. The mean PTA was 37.8 dB which was significantly decreased from the non-affected ear with PTA of 17.9 (*p* < 0.001). One patient developed vertiginous spells and unsteadiness 4 years after initial presentation and a repeat MRI revealed progression to utricular, saccular and cochlear hydrops. Vestibular testing was obtained in five patients with one patient presenting with 50% caloric paresis and all others normal. The most common treatment tried was acetazolamide in seven patients with 86% reporting subjective clinical improvement. Two out of the 10 patients had a history of migraine (20%).

**Conclusions:** Patients with MRI exhibiting isolated cochlear EH present with predominantly auditory symptoms: mild to moderate low-frequency hearing loss, aural fullness, tinnitus without significant vertigo. Isolated cochlear hydrops is more common in males, average age in mid-60's and there is a low comorbidity of migraine headaches. This contrasts significantly with patients with isolated saccular hydrops on MRI from our prior studies. We believe that isolated cochlear EH with hearing loss but no vertigo is distinct from Meniere's disease or its variant delayed endolymphatic hydrops. We propose that cochlear Meniere's disease represents a distinct clinical entity that could be a variant of Meniere's disease.

## Introduction

Meniere's disease is an inner ear disorder characterized with episodic spontaneous rotational vertigo, fluctuating hearing loss, tinnitus, and aural fullness. The diagnosis of Meniere's disease predominantly relies on clinical criteria as described by the 1995 American Academy of Otolaryngology—Head & Neck Surgery (AAO-HNS) and the 2015 Barany Society guidelines (1). Human temporal bone (HTB) studies have implicated dilation of the endolymphatic space—endolymphatic hydrops (EH)—as the nearly universal histopathological finding in Meniere's disease (2).

Cochlear hydrops or cochlear Meniere's disease was once classified as a distinct diagnostic entity, considered to be a variant of Meniere's disease without the vertigo. This group of patients previously diagnosed with cochlear Meniere's presented with a history of fluctuating auditory symptoms of aural fullness, tinnitus, and hearing loss without vertigo or vestibular symptoms (3). Despite the seemingly distinct clinical presentation of cochlear Meniere's, this entity was excluded by the Committee on Hearing and Equilibrium of the AAO-HNS in 1985 (4, 5). The histopathology of archival HTB studies of patients with a history of Meniere's disease, demonstrate that the pars inferior, the cochlea and saccule frequently display evidence of EH (6). However, it is important to evaluate for the presence and localization of EH during life in order to correlate with the clinical presentation. The question remains as to whether there is a specific histopathological correlate with isolated cochlear Meniere's.

High-resolution delayed contrast magnetic resonance imaging (MRI) has served to evaluate for endolymphatic hydrops *in vivo* to diagnose Meniere's disease and variants (7–9). High-resolution MRI imaging identification of EH involving the cochlea and vestibule is now achievable. In a study of eight patients with fluctuating hearing loss without vertigo, MRI evidence of cochlear and vestibular hydrops was identified in 100% of cases (10). A published case series of patients presenting with fluctuating aural pressure, tinnitus reported a response to medical therapy of diuretics and salt restriction in 80%, and evidence for hydrops using transtympanic electrocochleography (11). However, there has not been imaging evidence of the presence of isolated cochlear hydrops as a distinct clinical entity.

We report on a cohort of patients who display a unique set of clinical manifestations and demonstrate isolated cochlear hydrops. Importantly, little is clinically known about this entity. Herein, we aim to describe the clinical presentation of patients with isolated cochlear EH on MRI. We hypothesize that this entity is a clinically separate presentation of Meniere's disease, and we describe its clinical, audiometric, and MRI image features.

## Materials and Methods

### Patient Selection

The 1995 AAO-HNS clinical guidelines were used to assign a diagnosis for definite Meniere's disease (5). A retrospective inquiry of consecutive patients who presented with audiovestibular symptoms between July 2015 and August 2016 were included. Of these, those with MRI imaging demonstrating EH of the cochlea alone were included. Patients with a past history of temporal bone surgery were excluded. With Institutional Review Board approval, a waiver of Health Insurance Portability and Accountability Act authorization, and a waiver of informed consent, a database of patients imaged with delayed intravenous contrast-enhanced 3D-FLAIR MRI was reviewed (IRB 13-000089, GI).

### Data

Clinical histories, audiovestibular testing, and imaging from each patient were reviewed and recorded. Audiovestibular tests dated most closely with time of MRI were used for interpretation. The average follow-up was 16.5 months.

The pure tone average (PTA) was computed from frequencies 500, 1,000, 2,000 Hz as reported on the audiogram. Speech reception threshold (SRT) and word recognition scores (WRS) were also recorded.

Electronystagmography (ENG) testing was performed in five out of the 10 patients. Jonkee's formula was used to compute a unilateral canal paresis. A value >25% represented an abnormal response. Cervical vestibular-evoked myogenic potential (cVEMP) testing was performed in those undergoing ENG.

### MRI Technique

Images were acquired as previously described (9, 12–15). In brief, a 3-Tesla Skyra unit (Siemens, Erlangen, Germany) using a 16-channel head and neck coil paired with two 4-channel surface coils positioned over bilateral ears was used following a 4-hour delay of an intravenous injection of 0.2 mmol/kg of either gadobutrol (Gadavist), or gadobenate. Cisternographic heavily T2-weighted 3-D turbo spin echo sequence (sampling perfection with application-optimized contrasts by using different flip angle evolutions: T2 SPACE) and heavily T2-weighted 3-D FLAIR sequence (hT2w-FLAIR), and a 3-D FLAIR sequence with an inversion time of 2,050 ms, creating bright endolymph and dark perilymph were performed. A subtracted image was obtained with bright perilymph, dark endolymph, and intermediate-signal bone (9, 12, 13).

### Statistical Analysis

Data were tabulated into Microsoft Excel and analyzed using Stata 15.0 (College Station, TX). Audiometric data of the affected ear were compared to the contralateral, unaffected ear. A two-tailed paired student's *t*-test was performed to assess for differences in PTA, SRT, and WRS. In all instances, *p*-values < 0.05 were deemed significant.

## Results

### Demographic and Clinical Presentation

Ten patients with MRI evidence of isolated cochlear hydrops were identified and included in the study. These are cases of hydrops of the cochlear duct, but no hydrops of the saccule or the utricle. The mean age is 66.4 years with a range of 50–85 years. There were seven males (70%) and three females (30%). There was involvement of five left (50%) and five right (50%) inner ears. The mean follow-up was 16.5 months (range 1–48 months) and PTA for each patient is seen in Table 1.

**Table 1:** Pure tone average (PTA) of the affected ear and follow-up for each patient.

**Case**	**PTA (dB)**	**Follow-up (months)**
1	23	1
2	51	5
3	26	1
4	50	3
5	38	9
6	26	7
7	26	48
8	48	20
9	58.3	24
10	31.6	48

### Auditory Symptoms

Auditory symptoms were the predominant clinical features. Ninety percent reported hearing loss, 70% endorsed ipsilateral aural fullness, and 80% complained of ipsilateral tinnitus often described it as a “hissing” or “whooshing” sound (Table 2). Of nine patients who reported hearing loss, only one characterized the hearing loss as fluctuating, and occurring suddenly the following day after taking a higher-than-normal dose of tadalafil (Cialis) (15). This patient with SSNHL did not clinically improve despite oral prednisone, and a subsequent MRI identified isolated cochlear hydrops. Of note, this patient had a history of migraines however the SSNHL did not occur in the setting of a migraine. The remaining patients characterized their hearing loss as gradually worsening or stable. Audiometric data commonly showed a low-frequency SNHL pattern.

**Table 2:** Demographics and symptomatology; *utricular, saccular EH was identified on repeat MRI 4 years following initial presentation with isolated cochlear hydrops.

**Age**	**Years**
Mean +/– Std dev	66.4 +/– 11.4
Minimum	50
Maximum	85
**Pure tone average**	**dB**
Mean +/– Std dev	37.8 +/– 13.0
Minimum	23
Maximum	58.3
**Characteristic**	**Percentage (*n*)**
**Sex**	
Female	30% (3)
Male	70% (7)
**Affected ear**	
Left	50% (5)
Right	50% (5)
**Symptomatology**	
Aural fullness	70% (7)
Hearing loss	90% (9)
Tinnitus	80% (8)
Fluctuating hearing loss	10% (1)
Positional vertigo	10% (1)
Prolonged spontaneous vertigo	0%
Unsteadiness	10% (1)
Non-specific dizziness	10% (1)
**MRI evidence of hydrops**	
Cochlear	100% (10)
Utricular	11% (1)*
Saccular	11% (1)*
**Electronystagmography**	
Paresis or paralysis	10% (1)
Normal	40% (4)
Unknown	50% (5)
**Treatment**	
Acetazolomide	70% (7)
Hydrochlorothiazide	10% (1)
Betahistine	10% (1)
No treatment	10% (1)
**Mean follow-up (months)**	16.5; range 1–48

### Vestibular Symptoms

Vestibular symptoms were rare in these patients with isolated cochlear hydrops. No patient presented with prolonged spells of spontaneous rotational vertigo characteristic of Meniere's disease. One patient had a one-time occurrence of positional vertigo 1 month prior to the MRI imaging, and no further spells of vertigo in the ensuing subsequent 4 years of follow-up. One patient endorsed a sense of unsteadiness triggered by head positions, with a duration of several minutes and no further spells noted during follow-up.

### Cochlear Hydrops on MRI Testing

**Figure 1:**
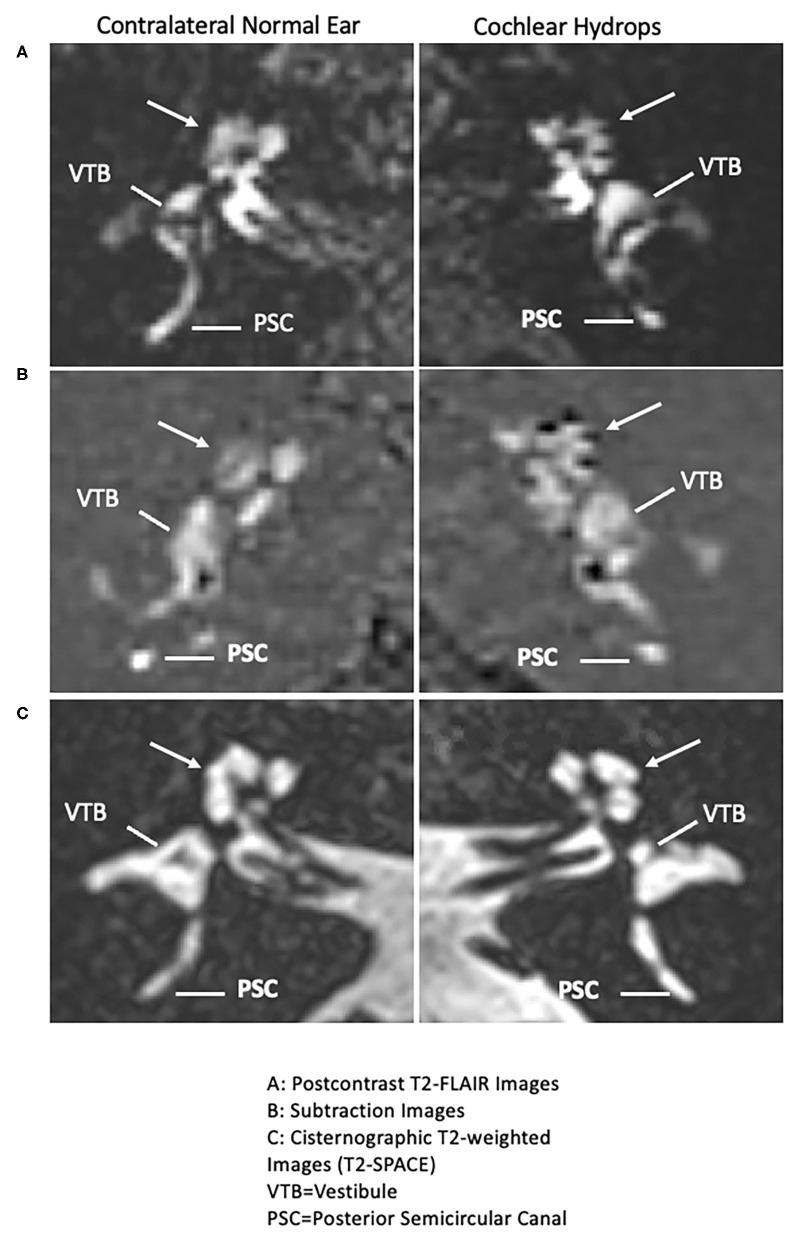
Isolated cochlear endolymphatic hydrops on 3T MRI: *Right* column: Cochlear hydrops and *Left* column: contralateral normal ear. **(A)** (Top right panel: Cochlear hydrops), delayed postcontrast T2-FLAIR images through the cochlea demonstrate a prominent scala media signal void consistent with a dilated cochlear duct (arrow) in the left ear (Top left panel: normal contralateral side), a normal appearing cochlea in the right ear (arrow). Posterior semicircular canal (PSC) and vestibule (VTB) are labeled for reference. **(B)** (Middle right panel: cochlear hydrops), corresponding subtracted images more clearly isolate the cochlear duct (arrow) delineated against a nullified background (Middle left panel: normal contralateral side), a normal appearing cochlea in the right ear (arrow). **(C)** Bottom right panel: Cochlear hydrops and Bottom left panel: normal contralateral side): both sides on corresponding cisternographic T2-weighted images (T2 SPACE), provided for anatomic reference, demonstrate normal fluid signal within the vestibule and cochlea on the disease side and the normal contralateral side, indicating isolated cochlear hydrops.

**Figure 2:**
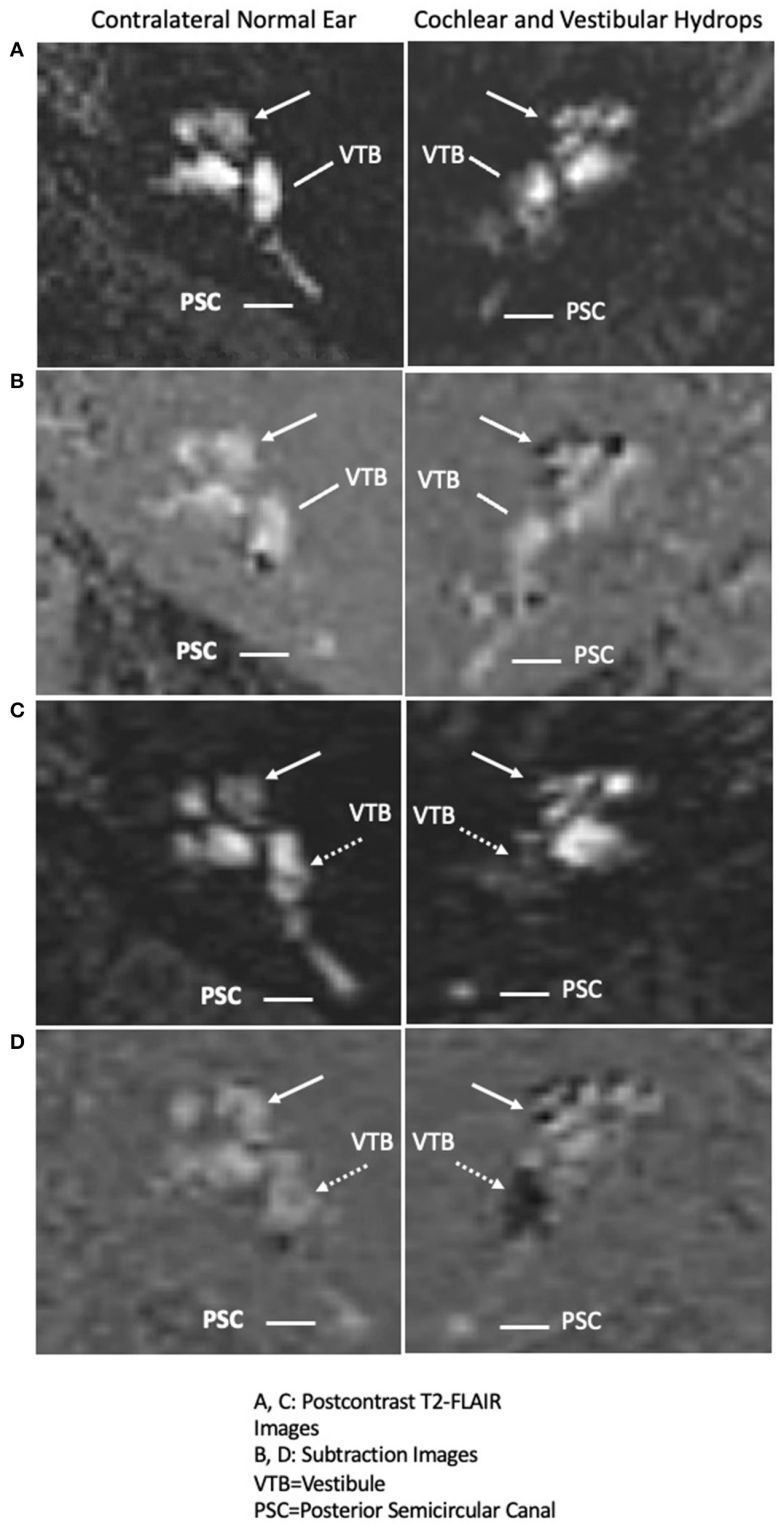
Interval progression of cochlear endolymphatic hydrops in 2015 to cochlear and vestibular endolymphatic hydrops in 2019 on 3T MRI: *Right* column: Endolymphatic hydrops and *Left* column: contralateral normal ear. **(A)** (Right panel: Cochlear hydrops in 2015 MRI), delayed postcontrast T2-FLAIR images through the cochlea demonstrate a prominent scala media signal void consistent with a dilated cochlear duct (arrow) in the right ear (Left panel: normal contralateral side), a normal appearing cochlea in the left ear. Posterior semicircular canal (PSC) and vestibule (VTB) are labeled for reference. **(B)** (Right panel: Cochlear hydrops in 2015 MRI), corresponding subtracted images more clearly isolate the cochlear duct delineated against a nullified background (Left panel: normal contralateral side), a normal appearing cochlea in the left ear. **(C)** (Right panel: Cochlear and Vestibular hydrops in 2019 MRI), delayed postcontrast T2-FLAIR images demonstrate a prominent cochlear duct and vestibule signal void consistent with cochlear (arrow) and vestibular (dashed arrow) hydrops in the right ear (Left panel: normal contralateral side), normal appearance of the cochlea and vestibule. **(D)** (Right panel: Cochlear and Vestibular hydrops in 2019 MRI), corresponding subtracted images more clearly isolate the cochlear duct (arrow) and vestibule (dashed arrow) against a nullified background in the right ear (Left panel: normal contralateral side), normal subtracted images of the cochlea and vestibule in the left ear.

All patients displaying evidence of cochlear hydrops with contrast-enhanced MRI of the internal auditory canal in the time span covering July 2015 until August 2016 were included. There were no temporal bone, internal auditory canal, or cerebellopontine angle lesions. Isolated cochlear hydrops as depicted by hT2w-FLAIR sequence is seen in Figure 1. Of note, one patient who demonstrated isolated cochlear hydrops in the first MRI developed utricular, saccule, and cochlear hydrops on repeat imaging 4 years after their initial presentation along with recurrent spells of vertigo and worsening hearing loss. The progression from isolated cochlear hydrops to utricular, saccular and cochlear hydrops on imaging 4 years later is seen in Figure 2.

### Comorbidity of Migraine

There were 2 (20%) patients with a comorbidity of migraine headaches as defined the International Headache Society criteria. No patients met the criteria for vestibular migraines. Both patients with a history of migraine were male. The patient who complained of non-specific unsteadiness had a history of migraine headaches, but there was no association of the migraine headaches with either the auditory or unsteadiness symptoms.

### Progression to Meniere's Disease in One Patient

One patient out of the 10 with unilateral cochlear hydrops exhibited progression to Meniere's disease 4 years later. At the time of the first MRI, he complained of long-standing unilateral aural fullness, tinnitus, and hearing loss. Approximately 4 years later, he developed worsening unilateral auditory symptoms and recurrent spells of vertigo and MRI revealed progression to utricular, saccular, and cochlear hydrops (Figure 2). There were no drop attacks in our cohort.

### Audiometry

**Figure 3:**
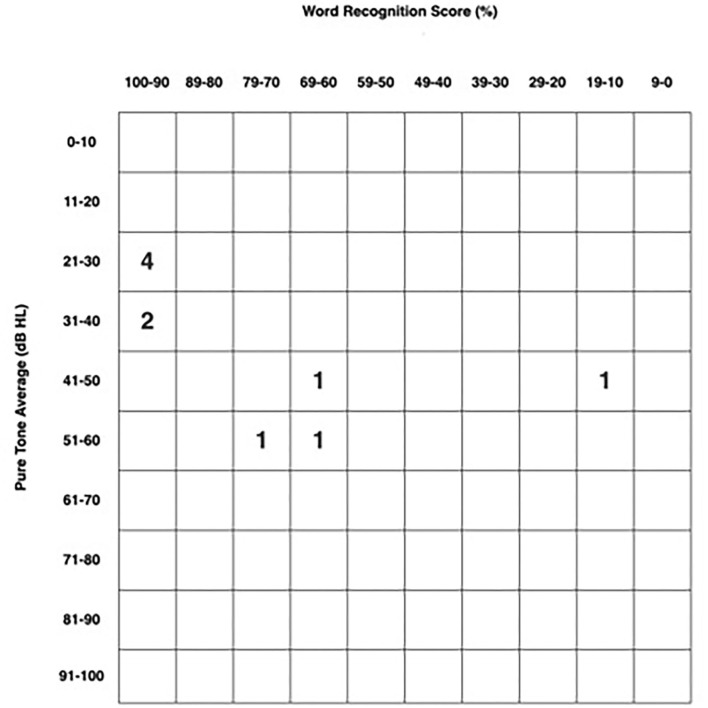
Standardized audiometric scattergram of 10 patients.

All patients had audiometric testing for review and are presented per the standardized hearing outcomes format as previously described in Gurgel et al. (14). One hundred percent of patients exhibited sensorineural patterns of hearing loss (Figure 3). Of these, 70% (seven of 10 patients) showed low-frequency, up-sloping SNHL, and 20% (two of 10 patients) showed a flat SNHL pattern and one patient (10%) had a down-sloping SNHL. The average PTA in the affected ear is 37.8 ± 13.0 dB (range 23–58.3 dB). Mean SRT was 36.0 ± 22.5 dB (range 10–80). Mean WRS in the affected ear were excellent at 80% (range 16–100%) and the median was 96%. The PTA in the affected ear (37.8 ± 13.0) was significantly worse than in the unaffected ear (17.9 ± 7.1) (*p* < 0.001). The SRT in the affected ear (36.0 ± 22.5) was significantly worse than in the unaffected ear (23.0 ± 16.9) (*p* = 0.001). The WRS in the affected ear (80.0 ± 27.5) was also significantly worse than in the unaffected ear (93.2 ± 15.3) (*p* = 0.02) (Table 3).

**Table 3:** Statistical analysis of audiometric data between affected and non-affected ear.

**Outcome**	**Mean (SD)**		***P*-value**
	**Affected ear**	**Non-affected ear**	
PTA	37.8 (13.0)	17.9 (7.1)	<0.001
SRT	36.0 (22.5)	23.0 (16.9)	0.001
WRS	80.0 (27.5)	93.2 (15.3)	0.02

*PTA, pure tone average; SRT, speech recognition threshold; WRS, word recognition score*.

### Vestibular Testing

Five of 10 patients underwent ENG testing. Caloric testing revealed one patient with 50% paresis on the ipsilateral side. This patient had one time episode of positional vertigo 1 month prior to the MRI but no subsequent vertigo. All other patients had normal caloric testing results. Vestibular-evoked myogenic potential responses were normal in all subjects.

## Discussion

Isolated cochlear hydrops on MRI has an apparent distinct clinical entity. All patients in the present study with isolated cochlear endolymphatic hydrops with no vestibular hydrops exhibited symptomatology of unilateral stable hearing loss, aural fullness, and tinnitus. The PTA ranged from mild to moderate low-frequency hearing loss (average 42 dB), with only one patient in the profound category. This contrasts to patients with sudden SNHL, which PTA may range between 58.9 and 86.4 dB and typically affected in multiple consecutive frequencies (15–17). In addition, patients with sudden SNHL typically have less recovery of hearing if higher frequencies are involved compared with sudden SNHL in the low frequencies. This may reflect a predominance of hydrops as causative of low frequency sudden SNHL, which may be more likely to fluctuate. The most common pattern of hearing loss in the cochlear hydrops cohort was a low frequency SNHL in eight out of 10 patients (80%), with one patient having a flat SNHL and one patient having a downsloping SNHL. Of note, the patient with a flat SNHL pattern had a low frequency hearing loss in an audiogram documented 2 years prior to the MRI at the onset of the auditory symptoms. In the cochlear hydrops cohort, low-frequency SNHL was common pattern with 85.7% clinically responding to acetazolamide with improvement in subjective tinnitus and hearing. However, in those who underwent repeat audiogram, the hearing loss remained stable.

Of interest, one of the 10 patients characterized the hearing loss as sudden in onset, with improvement using acetazolamide. While one of the patients had sudden hearing loss, this group of patients is distinct from the patients with the idiopathic sudden hearing loss which presents generally with an abrupt hearing loss without fluctuation. The temporal bones from patients with a history of sudden SNHL demonstrate findings of severe atrophy of the organ of Corti and tectorial membrane without endolymphatic hydrops (18). A recent study using high-resolution delayed contrast MRI imaging demonstrated that patients with sudden SNHL do not have endolymphatic hydrops (19).

Only one of the 10 patients reported vertigo in our study at the time of isolated cochlear hydrops on MRI. By description, this may have been a bout of benign paroxysmal positional vertigo. Another patient had a sense of unsteadiness predominantly with head movements. One other patient developed spells of vertigo 4 years later, and MRI revealed a progression to vestibular and cochlear hydrops. This patient is indicative of the potential for cochlear hydrops to present as a specific entity but may in some cases be early Meniere's disease.

The present study cohort have similar clinical presentations to a report by Nozawa et al. which identified prominent unilateral aural fullness, tinnitus, hearing loss in 50 patients with unilateral low-frequency SNHL. In their study, 33% described dizziness or unsteady sensation that was not fluctuating (20). Another separate retrospective study of 137 patients endorsed prominent unilateral auditory symptoms with approximately one-third unsteadiness or dizzy sensation immediately after rising (21). Both studies report a female predominance which differs from the present study of isolated cochlear hydrops which was 70% male. The clinical profile of symptoms is distinct from those of Meniere's disease characterized by spells of vertigo accompanied by reduced hearing, tinnitus, and aural fullness.

The management of the symptoms of isolated cochlear hydrops in this cohort was similar to that used for Meniere's disease. Notably, six out of 7 patients (85.7%) who were treated with acetazolamide reported significant improvement or resolution of symptoms. Of note, improvement in tinnitus and hearing was more commonly observed whereas aural fullness appeared to persist and respond poorly to medical management. One patient who clinically presented with tadalafil-related sudden SNHL with MRI evidence of isolated cochlear hydrops failed a course of prednisone, and subsequently had symptom improvement with acetazolamide (22). The rationale for using acetazolamide is based on an animal model where guinea pigs were administered oral acetazolamide and demonstrated reduced endolymphatic hydrops compared to those without treatment (23). Furthermore, patient's with Meniere's disease who respond to acetazolamide demonstrated reversal of endolymphatic hydrops with treatment based on MRI imaging (13).

There was one patient who presented with unilateral hearing loss, fullness, and tinnitus without vertigo had an MRI showing isolated cochlear hydrops, and would be diagnosed with cochlear Meniere's. Four years later, this patient had full spectrum Meniere's disease with recurrent spells of vertigo and unsteadiness and worsening auditory symptoms. The MRI progressed, consistent with ipsilateral cochlear, saccular, and utricular hydrops (Figure 2). This presentation has some overlapping features of delayed endolymphatic hydrops (DEH) which was first described by Nadol et al. (24). In DEH, episodic spells of vertigo occur 1–68 years following a preceding instance of sudden profound deafness (25). HTB studies have similarly identified EH on histopathologic analysis in DEH (25). However, this patient's initial hearing loss was mild at 26 dB. We speculate this patient progressed from cochlear EH to Meniere's disease, and that a subset of the isolated cochlear hydrops patients may represent an early form of Meniere's disease. Similarly, in our study of isolated saccular hydrops, one patient presented first with only sudden SNHL and then 3 years later developed full spectrum Meniere's disease with recurrent spells of room-spinning vertigo and the MRI demonstrated cochlear, saccular, and utricular hydrops (26).

It is particularly instructive to compare the present study cohort of patients with MRI demonstrating isolated cochlear hydrops to patients presenting with isolated saccular hydrops from our prior study. In the case of isolated saccular hydrops, the clinical presentation is the full spectrum of Meniere's disease with 12 out of 18 patients meeting criteria for Meniere's disease and four out of 18 meeting criteria for delayed endolymphatic hydrops (26). Thus, the presence of endolymphatic hydrops in the saccule in the case of sudden SNHL may be predictive of the development of Meniere's disease. Also, 22% of those with isolated saccular hydrops had a history of Tumarkin falls. This contrasts with the present study cohort of patients with isolated cochlear hydrops. There were no cases in the cochlear hydrops cohort meeting the criteria for Meniere's disease or delayed endolymphatic hydrops, and no cases of Tumarkin falls. In one patient, the isolated cochlear hydrops progressed to cochlear and vestibular hydrops with recurrent vertigo spells. Further studies may identify factors involved in progression of the endolymphatic hydrops.

In the present study of all patients with isolated cochlear hydrops, audiovestibular testing demonstrates a low-frequency hearing loss pattern in 80%, similar to saccular hydrops (83%) with a similar degree of hearing impairment with isolated saccular hydrops mean PTA 54 dB vs. isolated cochlear hydrops 37.8 dB and WRS 59 vs. 80%. In both isolated saccular hydrops and isolated cochlear hydrops, there is a history of unilateral tinnitus, aural fullness and subjective response to diuretic therapy. Furthermore, there is a distinction in the incidence of migraine headaches which was significantly lower in our cohort compared to saccular hydrops patients (22 vs. 61%). Demographically, it appears cochlear hydrops more often affects men (mean age 67.6 years, 78% male). However, females were the majority in isolated saccular hydrops (mean age 60.8 years, 61% female) (26). Larger studies are indicated to evaluate these demographic differences between patients with isolated cochlear hydrops and those with isolated saccular hydrops.

VEMP testing identified normal responses in the present study of isolated cochlear hydrops. It is possible that endolymphatic hydrops localizing only to the cochlea is associated with preservation of VEMP responses. A multivariable analysis of the VEMP responses noted that that EH of the vestibule had a greater effect on the decrement of VEMP responses than EH of the cochlea (27). Of the five patients in the cohort of cochlear hydrops who had vestibular testing, one patient had 50% ipsilateral caloric paresis presenting with one single spell of positional vertigo. In Gurkov et al., the degree of audiovestibular hydrops correlated with a progressive loss of auditory function, and a trend toward decrement of amplitude of VEMP (27). The presence of isolated saccular hydrops was indicative of Meniere's disease, and correspondingly 53% of those tested had a significant caloric paresis and 29% had reduced or absent VEMP responses (26).

In fifty-percent of our patients, an increased FLAIR signal in the perilymphatic space was noted in the inner ear with cochlear EH. In a previous study, our group attributed this radiographic finding to an increased blood-labyrinthine barrier permeability (28). Furthermore, extensive endothelial cell damage and the presence of oxidative stress markers were identified in a histopathologic analysis of human utricular macula from patients who underwent surgery for intractable Meniere's disease or delayed EH. These findings could represent a pathophysiologic mechanism or contribute to an increased permeability of the blood-labyrinthine barrier, and the subsequent development of EH (28, 29).

Migraine as a comorbidity was seen in only two of nine patients, and both were male with one patient endorsing non-specific dizziness or unsteadiness in addition to ipsilateral fluctuating mild hearing loss. Previous case studies reported a possible pathophysiologic link between migraine and sudden SNHL in a subset of idiopathic sudden hearing loss (30). There is one case report of MRI evidence of bilateral EH involving the vestibule and cochlea has been demonstrated in a patient with bilateral fluctuating hearing loss, tinnitus, without vertigo who may represent early bilateral Meniere's disease (31). Our cohort further contrasts with patients with Meniere's disease with migraine who frequently have bilateral subjective fluctuating hearing loss with a return to baseline normal hearing, and have an earlier age of symptom onset (32, 33). In the majority of cases of isolated cochlear hydrops, the symptoms were responsive to acetazolamide. The 20% comorbidity of migraines in the present study of isolated cochlear hydrops is not greater than the incidence in the general population. Additionally, in the two cases of comorbidity of migraines and cochlear hydrops, the migraines were not temporally associated with the inner ear symptoms. In contrast, Gurkov et al. study of 249 patients with MRI confirmed Meniere's disease, about 20% had migraine visual aura (phosphenes) simultaneous with the vertigo spells and more than half of the patients reported that headaches occurred in association with their vertigo (34).

There are limitations inherent to the current study. An element of selection bias exists as our cohort consists of patients who present to a tertiary university Neurotology clinic with hearing loss and or audiovestibular symptoms, and thus there is a high pre-test probability for endolymphatic hydrops. Further studies would be to conduct more extended follow-up to enable symptom tracking, treatment response, and the possibility to monitor for the evolution of symptoms and MRI findings.

## Data Availability Statement

The data analyzed in this study is subject to the following licenses/restrictions: they contain protected health information. Requests to access these datasets should be directed to jalonso@mednet.ucla.edu.

## Author Contributions

GI and AI: contributed to the conception and design of the project. JA: organized the database. JA and RF: performed the statistical analysis. NP and LL: contributed to the collection and refinement of radiologic images. All authors contributed to manuscript revision, read, and approved the submitted version.

## Conflict of Interest

The authors declare that the research was conducted in the absence of any commercial or financial relationships that could be construed as a potential conflict of interest.
